# The relationship between the ratio of non-high-density lipoprotein cholesterol to high-density lipoprotein cholesterol (NHHR) and both MASLD and advanced liver fibrosis: evidence from NHANES 2017–2020

**DOI:** 10.3389/fnut.2025.1508106

**Published:** 2025-02-27

**Authors:** Juyi Li, Chunjia Kou, Yuwei Chai, Yuchen Li, Xue Liu, Li Zhang, Haiqing Zhang

**Affiliations:** ^1^Department of Endocrinology, Shandong Provincial Hospital, Shandong University, Jinan, Shandong, China; ^2^Key Laboratory of Endocrine Glucose & Lipids Metabolism and Brain Aging, Ministry of Education, Jinan, Shandong, China; ^3^Department of Endocrinology, Geriatrics Center, The First Affiliated Hospital of Anhui University of Chinese Medicine, Hefei, Anhui, China; ^4^Department of Vascular Surgery, Shandong Provincial Hospital Affiliated to Shandong First Medical University, Jinan, Shandong, China; ^5^Department of Endocrinology, Shandong Provincial Hospital Affiliated to Shandong First Medical University, Jinan, Shandong, China; ^6^Shandong Key Laboratory of Endocrinology and Lipid Metabolism, Jinan, Shandong, China; ^7^Shandong Institute of Endocrine and Metabolic Diseases, Jinan, Shandong, China; ^8^Shandong Engineering Laboratory of Prevention and Control for Endocrine and Metabolic Diseases, Jinan, Shandong, China

**Keywords:** NHHR, MASLD, advanced liver fibrosis, NHANES, cross-sectional study

## Abstract

**Background:**

The non-HDL-C to HDL-C ratio (NHHR) is a dependable lipid marker linked to atherosclerotic traits. This study examines the potential relationship between NHHR and both metabolic dysfunction-associated steatotic liver disease (MASLD) and advanced liver fibrosis.

**Methods:**

This study investigated the relationship between NHHR levels and both MASLD and advanced liver fibrosis using data from the 2017–2020 National Health and Nutrition Examination Survey (NHANES) in the United States. First, we conducted a baseline characteristics analysis of the population based on NHHR quartiles. Second, we employed multivariable weighted linear regression models to examine the associations between NHHR and MASLD, as well as advanced liver fibrosis. Third, we utilized restricted cubic splines (RCS) to assess potential non-linear relationships. Fourth, we performed subgroup analyses. Finally, ROC curve analysis was conducted to evaluate the effectiveness of NHHR.

**Results:**

In the main analysis, this study included a total of 9,864 participants. Following multivariable logistic regression and comprehensive adjustments, elevated NHHR levels in the Q3 and Q4 groups were significantly linked to MASLD, with odds ratios of 1.59 (95% CI: 1.20–2.11) and 1.83 (95% CI: 1.40–2.39), respectively (*P* for trend < 0.0001). Elevated NHHR levels in the Q2 and Q3 groups remained significantly linked to a decreased risk of advanced liver fibrosis, with odds ratios of 0.61 (95% CI 0.40–0.94, *P* = 0.03) and 0.64 (95% CI 0.47–0.89, *P* = 0.01), respectively. RCS analysis revealed a U-shaped nonlinear association between NHHR and both MASLD (*P* = 0.000; *P* for nonlinear = 0.029) and advanced liver fibrosis (*P* = 0.0001; *P* for nonlinear = 0.000). In the subgroup analysis, we found that this relationship was significant only in certain subgroups. The ROC curve analysis revealed that NHHR exhibited the best predictive performance for diagnosing MASLD based on the fatty liver index (FLI). The optimal cutoff point for NHHR in predicting MASLD using FLI was determined to be 2.476, with sensitivity and specificity values of 0.589 and 0.698, respectively.

**Conclusion:**

NHHR may serve as a predictive marker for MASLD and advanced liver fibrosis, highlighting its potential significance in risk assessment and prevention strategies.

## Introduction

The global prevalence of metabolic dysfunction-associated steatotic liver disease (MASLD) has risen in recent years, paralleling the increasing incidence of metabolic disorders (1, 2). It is estimated that approximately 1 billion individuals worldwide have MASLD (2). We have noticed that MASLD emphasizes the critical role of metabolic factors and refines the impact of factors such as viral infections and alcohol on diagnosis (3). MASLD, as a prevalent condition, is closely linked to the rising incidence of metabolic-related diseases such as obesity and diabetes, significantly impacting global health.

Among these, a subtype of MASLD characterized by inflammation is a progressive liver condition that can result in fibrosis and may eventually advance to cirrhosis or hepatocellular carcinoma (4). Cirrhosis and related cancers significantly increase the mortality associated with liver diseases, imposing a substantial health and economic burden on patients and society. Therefore, monitoring the progression of MASLD is crucial. Currently, the only reliable diagnostic tool is liver biopsy, which is limited by its invasiveness, poor acceptability, sampling errors, and high cost (5).

At present, The increasing obesity rates are contributing to a growing population with metabolic disorders, which can result in metabolic syndrome, marked by abnormal glucose levels, insulin resistance, hypertension, and dyslipidemia (6). Dyslipidemia is marked by increased non-high-density lipoprotein cholesterol (non-HDL-C) and triglycerides (TG), and reduced high-density lipoprotein cholesterol (HDL-C) levels. There is a scarcity of literature that thoroughly explores the interrelationships between metabolic disease-related indicators and MASLD. Also, researches suggested that the non-HDL-C/HDL-C ratio (NHHR) is an effective predictor of metabolic syndrome (6–8). This provides a rationale for selecting this parameter as a research indicator. However, to our knowledge, no studies have extensively examined whether NHHR can act as a predictor for MASLD and liver cirrhosis. For patients, earlier or less invasive diagnostics can facilitate the early identification of at-risk individuals, significantly improving patient outcomes. Therefore, this study utilizing a large-scale, representative sample, to explore the association between NHHR and both MASLD and cirrhosis, as well as assess its predictive value for these conditions. The integration of NHHR evaluation into clinical practice represents a promising advancement in the management of MASLD and cirrhosis. By facilitating earlier and less invasive diagnoses, NHHR can play a pivotal role in improving patient care and outcomes in the context of liver diseases.

## Materials and methods

### Study population

**FIGURE 1:**
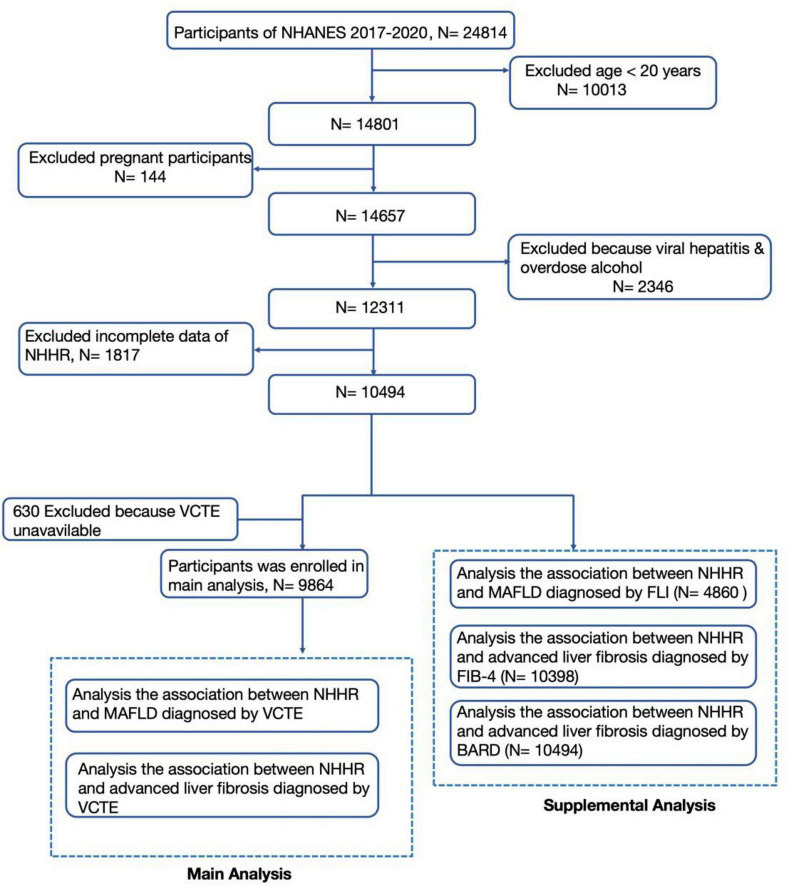
Study flowchart.

The National Health and Nutrition Examination Survey (NHANES) is an extensive and continuous program. It is conducted by the National Center for Health Statistics (NCHS) under the Centers for Disease Control and Prevention (CDC). For our analysis, we collected and analyzed data from the 2017–2018 NHANES cycle. Among the 24,814 participants from the NHANES study conducted between 2017 and 2020, 14,657 were non-pregnant adults aged 20 and older. After excluding 2,346 individuals with viral hepatitis and those who had excessive drinking, we retained 12,311 participants. Viral hepatitis was diagnosed through a positive hepatitis B surface antigen (HBsAg) test. It can also be identified by the presence of hepatitis C virus (HCV) RNA. High alcohol consumption is characterized by a daily intake of at least three drinks for women. For men, it is defined as a daily intake of four drinks. Additionally, consuming five or more drinks on a single occasion within a month also indicates high alcohol consumption (9). Participants with incomplete NHHR data were excluded, resulting in 10,494 individuals eligible for further selection. The primary analysis included 9,864 individuals after excluding 630 participants lacking VCTE data. For the supplementary analysis investigating the relationship between NHHR and MASLD diagnosis using the fatty liver index (FLI), 4,860 participants were enrolled. The evaluation of the association between NHHR and advanced liver fibrosis, based on fibrosis-4 index (FIB-4) and BARD criteria, included 10,398 and 10,494 participants, respectively (Figure 1). The NCHS Ethics Review Board approved NHANES 2017–2020, and all participants provided informed consent. Data collection and analysis were undertaken in accordance with NHANES guidelines.

### Assessment of MASLD and advanced liver fibrosis assessments

Liver stiffness and CAP, important indicators of liver steatosis, were assessed using VCTE with the FibroScan 502 V2 Touch device (Echosens, North America). The device at the NHANES Mobile Examination Center (MEC) was fitted with either a medium (M) or extra-large (XL) probe. FLI was ranged from 0 to 100 using the followed formula: (e^0.953*loge (*triglycerides*)^ ^+0.139*BMI+0.718*loge (*ggt*)+0.053*waist circumference–15.745^)/(1+e^0.953*loge (*triglycerides*)+0.139*BMI+0.718*loge (*ggt*)+0.053*waist circumference–15.745^) ∗ 100 (10). FIB-4 was calculated by followed formula: age (years) × AST [U/L] / (platelets [10^9^/L] × (ALT [U/L])1/2) (10). In contrast, the BARD score, which ranges from 0 to 4, is based on BMI, the AST/ALT ratio, and a history of diabetes. A BMI above 28 kg/m^2^ earns 1 point, an AST/ALT ratio greater than 0.8 gives 2 points, and a history of diabetes adds another point. MASLD was defined as the coexistence of hepatic steatosis and at least one of the five cardiometabolic criteria (11): (1) BMI ≥ 25 kg/m^2^ or waist circumference ≥ 94 cm for males and ≥ 80 cm for females, (2) fasting glucose ≥ 100 mg/dl or 2-h post-load glucose levels ≥ 140 mg/dl or hemoglobin A1c ≥ 5.7% or diabetes mellitus or treatment for diabetes mellitus, (3) blood pressure ≥ 130/85 mmHg or antihypertensive drug treatment, (4) fasting plasma triglycerides ≥ 150 mg/dl or lipid-lowering treatment, (5) plasma HDL-cholesterol < 40 mg/dl for men and < 50 mg/dl for women or lipid-lowering treatment. Hepatic steatosis diagnosis requires meeting these criteria: (1) liver steatosis (CAP ≥ 248 DB/m) (12); (2) FLI ≥ 60 (13). For the identification of advanced liver fibrosis, three conditions are necessary: (1) VCTE ≥ 8.8 E/kPa (indicating liver stiffness F3 and F4) (14), (2) FIB-4 > 3.25 (15), and (3) BARD ≥ 2 (10, 16).

### Assessment of NHHR

The NHHR functions as an independent variable for assessing exposure. To compute NHHR, we utilized the Non-HDL-C/HDL-C ratio method established in previous studies (17). Non-HDL-C is measured by subtracting HDL-C from total cholesterol (TC) using the lipid profiles of fasting subjects. The levels of TC and HDL-C were detected by automatic biochemical analyzer. For determining TC concentrations, both the Roche Cobas 6000 and Roche Modular *P* chemical analyzers were employed during the analytical process.

### Study variables

We analyzed sociodemographic factors including gender, age, education level (categorized as below high school, high school or equivalent, and above high school), and race/ethnicity (classified as Mexican American, non-Hispanic Black, non-Hispanic White, other Hispanic, and other races). We examined the poverty-income ratio (PIR), assessing family income against the poverty threshold. For analytical purposes, we divided PIR into three categories: less than 1.30, 1.30 to 3.49, and 3.50 or higher. This framework allowed us to examine the potential effects of sociodemographic factors on our results. We also evaluated health-related covariates such as body mass index (BMI in kg/m^2^), waist circumference (WC in cm), smoking status, alcohol use, and physical activity levels. BMI is calculated by dividing a person’s weight in kilograms by their height in meters squared. Smoking status was classified as follows: individuals who had never smoked 100 cigarettes were considered never smokers. Individuals were classified as former smokers if they answered “No” to the current smoking question, and as current smokers if they responded “Yes” (18). Alcohol consumption was categorized into three groups: “never” drinkers (fewer than 12 drinks per year), “former” drinkers (previously consumed 12 or more drinks annually but have stopped), and “current” drinkers (consume 12 or more drinks annually) (18). Moderate drinking is characterized by a daily intake of at least two drinks for women and three for men, or engaging in binge drinking twice or more monthly (9).

Participants were categorized based on their weekly metabolic equivalent (MET) minutes of vigorous activity into four groups: No moderate to vigorous physical activity (NMVPA) for 0 MET-minutes/week, Low (LMVPA) for 1–599 MET-minutes/week, Moderate (MMVPA) for 600–1,199 MET-minutes/week, and High (HMMVPA) for 1,200 or more MET-minutes/week.

Venous blood samples were collected to assess various biomarkers, including levels of alanine aminotransferase (ALT) and aspartate aminotransferase (AST). Based on fasting blood glucose and insulin levels, we evaluated insulin resistance using the homeostasis model assessment of insulin resistance (HOMA-IR). The formula for HOMA-IR is:


$$H\,O\,M\,A-I\,R\,\frac{I\,n\,s\,u\,l\,i\,n\,\left(\mu \,U/m\,l\right)\,G\,l\,u\,c\,o\,s\,e\,\left(m\,m\,o\,l/L\right)}{22.5}$$

We also assessed the presence of various coexisting conditions, particularly Diabetes Mellitus (DM) and hypertension. Diabetes diagnosis was determined by a glycohemoglobin level of at least 6.5%, the use of diabetes medications or insulin, or a self-reported diabetes diagnosis (19). Hypertension was defined by criteria such as a mean systolic blood pressure ≥ 140 mmHg, a mean diastolic blood pressure ≥ 90 mmHg, or a self-reported hypertension diagnosis (20).

### Statistical analysis

We utilized the “mice” package to create five imputed datasets through chained equations for addressing missing covariate data. This method was designed to minimize the effect of missing data on our analyses. Post-imputation, we evaluated the robustness of our findings through sensitivity analyses across five data sets (refer to Supplementary Tables 1, 2).

Throughout the study, we utilized a variety of strategies to reduce duplicate information. Baseline characteristics by NHHR quartiles were presented using weighted means and standard errors for continuous variables, and weighted proportions for categorical variables. By incorporating sampling weights, the population is represented more accurately.

We employed multivariable weighted linear regression models to examine the association between NHHR and both MASLD and advanced liver fibrosis diagnosed through VCTE, allowing for an analysis of NHHR’s influence on these conditions while adjusting for pertinent factors. Restricted cubic splines were employed to assess non-linear relationships by modeling complex patterns deviating from linearity. In addition, threshold effect analyses were performed to identify significant cut-off points. Data were stratified by various factors for subgroup analyses to evaluate how the association between NHHR and body fat distribution varies among different groups. To assess NHHR’s effectiveness, we examined ROC curves to depict sensitivity against 1-specificity and determine cut-off points from the results. Our approach seeks to provide a comprehensive analysis, minimizing redundancies and highlighting critical nuances in the relationship between NHHR, MASLD, and advanced liver fibrosis. A *p*-value below 0.05 was deemed statistically significant. All analyses were conducted using R software (version 4.4.1).

## Results

### Baseline characteristics of participants stratified by NHHR quartiles

**FIGURE 2:**
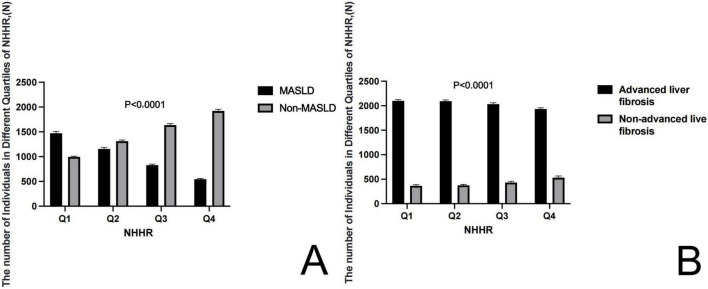
The box graph shows the number of individuals in the quartiles of NHHR groups. **(A)** Distribution of individuals across NHHR quantiles in MASLD and Non-MASLD groups. **(B)** Distribution of individuals across NHHR quantiles in advanced liver fibrosis and Non-advanced liver fibrosis groups.

The main analysis of this study involved 9,864 participants, divided into four groups according to quartiles of NHHR. Specifically, 2,469 participants were assigned to Group Q1 (48.62–109.24), 2,461 to Group Q2 (109.24–135.57), 2,468 to Group Q3 (135.57–168.38), and 2,466 to Group Q4 (168.38–1,075.92). Table 1 displays the baseline characteristics of participants categorized by NHHR strata. We analyzed baseline characteristics across the NHHR quartiles, finding significant differences in age, gender, race, education, smoking status, alcohol consumption, physical activity, BMI, waist circumference, ALT levels, HOMA-IR, and the prevalence of Diabetes Mellitus and hypertension. We conducted a stratified analysis based on MASLD presence and advanced liver fibrosis diagnosed through VCTE to explore potential NHHR differences among the groups. We compared NHHR values concerning the presence of MASLD and advanced liver fibrosis, diagnosed via vibration-controlled transient elastography. Figure 2 illustrates significant differences in NHHR between the MASLD and non-MASLD groups, as well as between individuals with and without advanced liver fibrosis.

**TABLE 1:** Characteristics of enrolled participants based on the NHHR quartile.

Variable	Total	Q1 [48.62–109.24]	Q2 [109.24–135.57]	Q3 [135.57–168.38]	Q4 [168.38–1,075.92]	*P*-value
Age (years)	50.08 (0.44)	49.56 (0.78)	50.18 (0.60)	51.78 (0.51)	48.78 (0.57)	< 0.001*
**Sex**						< 0.0001*
Male	4,692 (47.42)	910 (32.88)	998 (39.34)	1,229 (51.98)	1,555 (64.80)	
Female	5,172 (52.58)	1,559 (67.12)	1,463 (60.66)	1,239 (48.02)	911 (35.20)	
**Race**						< 0.0001*
Mexican American	1,115 (7.45)	219 (6.45)	265 (7.11)	296 (7.25)	335 (8.95)	
Non-Hispanic Black	2,465 (11.20)	780 (14.65)	676 (12.50)	568 (9.89)	441 (7.90)	
Non-Hispanic White	3,407 (63.52)	836 (62.98)	880 (64.22)	856 (64.33)	835 (62.55)	
Other Hispanic	977 (7.17)	185 (6.52)	236 (6.56)	240 (6.53)	316 (9.03)	
Other race	1,900 (10.66)	449 (9.39)	404 (9.61)	508 (12.00)	539 (11.57)	
**Education levels**						< 0.001*
Under high school	809 (3.74)	144 (2.80)	171 (2.94)	246 (4.33)	248 (4.85)	
High school or equivalent	3,300 (33.38)	841 (30.57)	768 (31.49)	818 (33.89)	873 (37.52)	
College graduate or above	5,738 (62.81)	1,480 (66.63)	1,519 (65.57)	1,400 (61.78)	1,339 (57.63)	
**Poverty income ratio**						0.21
< 1.30	2,625 (19.30)	651 (17.47)	600 (18.06)	710 (21.29)	664 (20.29)	
1.30–3.49	3,904 (34.39)	949 (35.38)	1,016 (36.07)	947 (33.01)	992 (33.16)	
**≥** 3.50	3,335 (46.31)	869 (47.15)	845 (45.87)	811 (45.71)	810 (46.54)	
**Smoke status**						0.001*
Never	6,057 (61.03)	1,558 (65.35)	1,553 (59.79)	1,507 (62.70)	1,439 (56.44)	
Former	2,410 (26.36)	564 (23.50)	607 (28.75)	602 (23.25)	637 (29.86)	
Now	1,395 (12.61)	345 (11.15)	301 (11.46)	359 (14.05)	390 (13.70)	
**Alcohol status**						< 0.0001*
Never	1,039 (8.16)	220 (8.90)	256 (9.52)	305 (13.84)	258 (10.99)	
Mild	4,025 (45.17)	1,001 (55.46)	967 (57.05)	997 (60.02)	1,060 (66.42)	
Moderate	1,868 (22.29)	542 (35.65)	516 (33.43)	408 (26.13)	402 (22.59)	
**Physical activity**						< 0.001*
NMVPA	2,538 (21.06)	629 (20.27)	593 (19.58)	648 (19.79)	668 (24.54)	
LMVPA	1,247 (11.58)	251 (8.03)	344 (11.80)	339 (14.59)	313 (11.75)	
MMVPA	1,011 (10.26)	254 (10.02)	240 (10.33)	260 (11.51)	257 (9.18)	
HMVPA	5,068 (57.10)	1,335 (61.68)	1,284 (58.29)	1,221 (54.10)	1,228 (54.52)	
**BMI**						< 0.0001*
Under weight	141 (1.54)	86 (3.56)	29 (1.13)	14 (1.17)	12 (0.38)	
Normal weight	2,397 (24.78)	996 (43.45)	645 (28.14)	447 (17.65)	309 (10.69)	
Over weight	3,226 (32.16)	737 (28.81)	821 (33.23)	831 (35.66)	837 (30.79)	
Obesity	4,100 (41.53)	650 (24.19)	966 (37.50)	1,176 (45.53)	1,308 (58.14)	
WC (cm)	100.71 (0.41)	92.35 (0.35)	98.90 (0.50)	103.47 (0.57)	107.74 (0.70)	< 0.0001*
ALT (IU/L)	22.08 (0.17)	18.71 (0.30)	20.35 (0.41)	22.76 (0.31)	26.37 (0.52)	< 0.0001*
AST (IU/L)	21.49 (0.15)	21.60 (0.31)	21.01 (0.38)	20.87 (0.27)	22.47 (0.29)	0.01*
HDL (mmol/L)	1.38 (0.01)	1.77 (0.01)	1.46 (0.01)	1.26 (0.01)	1.06 (0.01)	< 0.0001*
Non-HDL (mmol/L)	3.49 (0.03)	2.50 (0.03)	3.16 (0.02)	3.67 (0.03)	4.60 (0.03)	< 0.0001*
HOMA IR	4.72 (0.11)	2.97 (0.13)	4.59 (0.30)	5.09 (0.16)	6.15 (0.25)	< 0.0001*
**DM**						< 0.0001*
Yes	2,111 (16.02)	459 (12.61)	505 (14.99)	545 (16.54)	602 (19.80)	
No	7,753 (83.98)	2,010 (87.39)	1,956 (85.01)	1,923 (83.46)	1,864 (80.20)	
**Hypertension**						< 0.0001*
Yes	4,393 (37.44)	1,040 (30.84)	1,088 (37.14)	1,126 (40.79)	1,139 (40.77)	
No	5,464 (62.52)	1,428 (69.16)	1,372 (62.86)	1,340 (59.21)	1,324 (59.23)	

*P < 0.05.

### Significant association between NHHR levels and MASLD: insights from logistic regression analysis

In the main analysis, multiple logistic regression was employed to evaluate the independent association between NHHR and MASLD. Model 1 did not involve any adjustments. Higher NHHR levels in the Q2, Q3, and Q4 groups were significantly associated with increased odds of MASLD, with odds ratios of 1.70 (95% CI 1.48–1.96), 3.14 (95% CI 2.70–3.66), and 5.26 (95% CI 4.50–6.16), respectively, compared to the Q1 group (*P* for trend < 0.0001). Model 2 was adjusted for age, race, gender, PIR, education level, physical activity, BMI, waist circumference, smoking status, and alcohol consumption. After adjustments, elevated NHHR levels in Q2, Q3, and Q4 groups remained significantly associated with MASLD, with odds ratios of 1.11 (95% CI: 0.83–1.49), 1.65 (95% CI: 1.27–2.12), and 1.99 (95% CI: 1.56–2.54), respectively (*P* for trend < 0.0001). To further account for additional covariates, Model 3 included adjustments for age, sex, education, race, PIR, physical activity, BMI, waist circumference, smoking status, alcohol consumption, ALT, AST, HOMA-IR, Diabetes Mellitus, and hypertension. After adjustments, elevated NHHR levels in Q3 and Q4 groups were significantly linked to MASLD, with odds ratios of 1.59 (95% CI: 1.20–2.11) and 1.83 (95% CI: 1.40–2.39), respectively, and a trend *P*-value of < 0.0001 (Table 2).

**TABLE 2:** Association between NHHR and MASLD diagnosed by vibration controlled transient elastography.

	Model 1		Model 2		Model 3	
	**OR (95% CI)**	***P*-value**	**OR (95% CI)**	***P*-value**	**OR (95% CI)**	***P*-value**
NHHR	1.01 (1.01, 1.02)	< 0.0001*	1.01 (1.00, 1.01)	< 0.0001*	1.01 (1.00, 1.01)	< 0.001*
Q1	Ref	Ref	Ref	Ref	Ref	Ref
Q2	1.70 (1.48, 1.96)	< 0.0001*	1.11 (0.83, 1.49)	0.46	1.06 (0.78, 1.44)	0.70
Q3	3.14 (2.70, 3.66)	< 0.0001*	1.65 (1.27, 2.12)	< 0.001*	1.59 (1.20, 2.11)	0.004*
Q4	5.26 (4.50, 6.16)	< 0.0001*	1.99 (1.56, 2.54)	< 0.0001*	1.83 (1.40, 2.39)	< 0.001*
***P*** for trend		< 0.0001*		< 0.0001*		< 0.0001*

Model 1: Non-adjusted. Model 2: Adjusted for age, sex, education level, race, PIR, PA, BMI, WC, smoke status and alcohol status. Model 3: Adjusted for age, sex, education level, race, PIR, PA, BMI, WC, smoke status, alcohol status, ALT, AST, TG, LDL, HOMA-IR, DM and hypertension.

*p < 0.05.

In the secondary analysis, we redefined MASLD based on serum levels of FLI to further explore its correlation with NHHR (Supplementary Table 3). Compared to diagnoses made with vibration-controlled transient elastography, higher NHHR levels in the Q3 and Q4 groups maintained a significant positive association with MASLD across all models: Model 1 (OR = 3.48, 95% CI: 2.63–4.59; OR = 6.49, 95% CI: 4.68–9.00; *P* for trend < 0.0001), Model 2 (OR = 4.65, 95% CI: 2.96–7.32; OR = 13.32, 95% CI: 7.53–23.54; *P* for trend < 0.0001), and Model 3 (OR = 3.90, 95% CI: 2.46–6.17; OR = 11.66, 95% CI: 6.56–20.72; *P* for trend < 0.0001).

### Significant association between NHHR levels and advanced liver fibrosis: insights from logistic regression analysis

In the main analysis, multiple logistic regression was utilized to assess the independent association between NHHR and advanced liver fibrosis. In Model 2, adjustments were made for age, gender, education level, race, physical activity, PIR, BMI, smoking status, waist circumference, and alcohol consumption. Higher NHHR levels in the Q2 and Q3 groups were significantly associated with advanced liver fibrosis compared to the reference group, with odds ratios of 0.60 (95% CI 0.41–0.89, *P* = 0.01) and 0.60 (95% CI 0.43–0.83, *P* = 0.004), respectively. To further account for additional covariates, Model 3 included adjustments for age, sex, education, race, PIR, physical activity, BMI, waist circumference, smoking status, alcohol consumption, ALT, AST, HOMA-IR, Diabetes Mellitus, and hypertension. In this model, elevated NHHR levels in the Q2 and Q3 groups were significantly linked to a lower risk of advanced liver fibrosis, with odds ratios of 0.61 (95% CI 0.40–0.94, *P* = 0.03) and 0.64 (95% CI 0.47–0.89, *P* = 0.01), respectively (Table 3).

**TABLE 3:** Association between NHHR and advanced liver fibrosis.

	Model 1		Model 2		Model 3	
	**OR (95% CI)**	***P*-value**	**OR (95% CI)**	***P*-value**	**OR (95% CI)**	***P*-value**
NHHR	1.00 (1.00, 1.01)	< 0.001*	1.00 (1.00, 1.00)	0.16	1.00 (1.00, 1.01)	0.49
Q1	Ref	Ref	Ref	Ref	Ref	Ref
Q2	0.87 (0.66, 1.16)	0.34	0.60 (0.41, 0.89)	0.01*	0.61 (0.40, 0.94)	0.03*
Q3	1.11 (0.83, 1.48)	0.47	0.60 (0.43, 0.83)	0.004*	0.64 (0.47, 0.89)	0.01*
Q4	1.60 (1.28, 2.01)	< 0.001*	0.82 (0.57, 1.18)	0.27	0.81 (0.54, 1.21)	0.28
***P*** for trend		< 0.0001*		0.586		0.564

Model 1: Non-adjusted. Model 2: Adjusted for age, sex, education level, race, PIR, PA, BMI, WC, smoke status and alcohol status. Model 3: Adjusted for age, sex, education level, race, PIR, PA, BMI, WC, smoke status, alcohol status, ALT, AST, HOMA-IR, DM and hypertension.

*p < 0.05.

In the secondary analysis, we redefined advanced liver fibrosis utilizing serum levels of FIB-4 and BARD to further examine its correlation with NHHR (Supplementary Tables 4, 5). For the FIB-4 metric, elevated NHHR levels in the Q3 group were significantly positively associated with MASLD in all models: Model 1 (OR = 0.20, 95% CI 0.11–0.36, *P* < 0.0001), Model 2 (OR = 0.20, 95% CI 0.08–0.51, *P* = 0.002), and Model 3 (OR = 0.32, 95% CI 0.13–0.80, *P* = 0.02). In the BARD analysis, when NHHR was considered a continuous variable in Model 3, it continued to serve as an independent protective factor against advanced liver fibrosis (OR = 0.85, 95% CI: 0.77–0.94, *P* = 0.004), compared to diagnoses based on vibration-controlled transient elastography.

### U-shaped nonlinear relationship between NHHR and MASLD/advanced liver fibrosis: evidence from restricted cubic splines analysis

**FIGURE 3:**
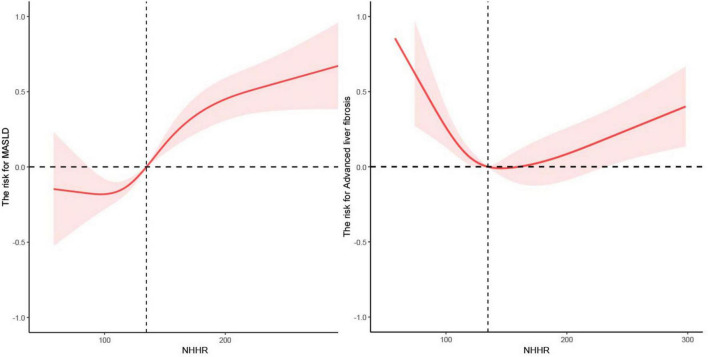
The nonlinear relationship between NHHR with MASLD and advanced liver fibrosis.

Utilizing restricted cubic splines and adjusting for age, education level, sex, race, PIR, physical activity, BMI, waist circumference, smoking status, alcohol consumption, ALT, AST, HOMA-IR, Diabetes Mellitus, and hypertension, we identified a U-shaped nonlinear relationship between NHHR and both MASLD (*P* for all = 0.000; *P* for nonlinear = 0.029) (see Figure 3) and advanced liver fibrosis (*P* for all = 0.0001; *P* for nonlinear = 0.000) (see Figure 3), indicating that both low and high NHHR values are associated with an increased risk of advanced fibrosis, while moderate NHHR values may be associated with a lower risk.

### Subgroup analysis reveals differential impact of NHHR on MASLD and advanced liver fibrosis

**FIGURE 4:**
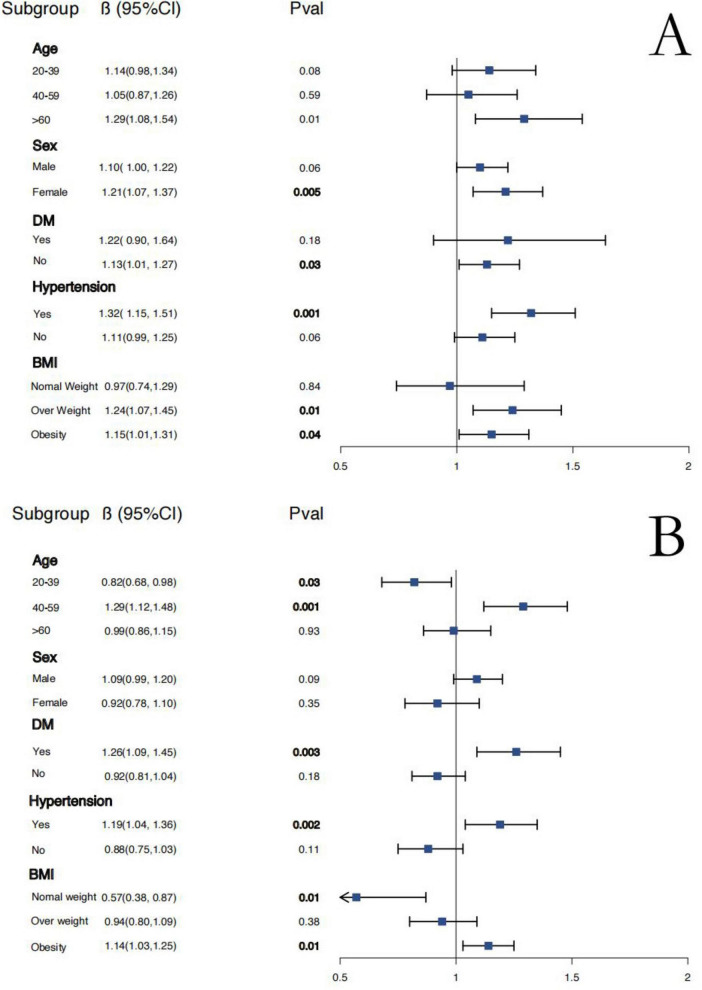
The relationship between NHHR with MASLD **(A)** and advanced liver fibrosis **(B)** in subgroups.

Additionally, subgroup analyses were conducted based on age, sex, Diabetes Mellitus, hypertension, and BMI to explore the independent association between NHHR, MASLD, and advanced liver fibrosis diagnosed via VCTE. The findings revealed that NHHR was a significant independent risk factor for MASLD in specific groups, including older adults (over 60), females, individuals without diabetes, those with hypertension, and overweight or obese participants (see Figure 4A). In contrast, NHHR served as a significant independent protective factor against advanced liver fibrosis primarily among younger and normal-weight individuals. Conversely, in middle-aged participants, as well as those with diabetes, hypertension, and obesity, NHHR emerged as an independent risk factor for advanced liver fibrosis (see Figure 4B).

### ROC curve analysis identifies optimal NHHR cutoff points for predicting MASLD and advanced liver fibrosis

**FIGURE 5:**
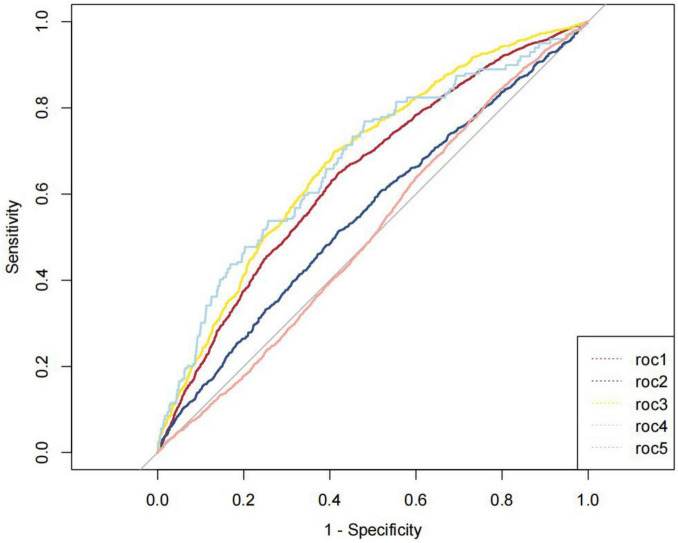
ROC curves for optimal cut-points of NHHR.

Figure 5 illustrates the areas under the curve (AUC) for MASLD diagnosed via VCTE (roc1), advanced liver fibrosis via VCTE (roc2), MASLD using FLI (roc3), advanced liver fibrosis assessed by FIB-4 (roc4), and advanced liver fibrosis using BARD (roc5), which were 0.643 (95% CI: 0.632–0.654), 0.554 (95% CI: 0.538–0.569), 0.680 (95% CI: 0.664–0.695), 0.677 (95% CI: 0.637–0.716), and 0.510 (95% CI: 0.499–0.521), respectively. The diagnostic capability of NHHR for predicting MASLD using FLI surpasses that of MASLD via VCTE, advanced liver fibrosis via VCTE, advanced liver fibrosis assessed by FIB-4, and advanced liver fibrosis using BARD. The optimal cutoff point for NHHR in predicting MASLD using FLI was found to be 2.476, with sensitivity and specificity values of 0.589 and 0.698, respectively.

## Discussion

Previous cross-sectional studies have identified the non-HDL-C/HDL-C ratio (NHHR) as one of the risk factors for metabolic-associated fatty liver disease (MAFLD) (21). However, no current research has demonstrated the association of NHHR with metabolic dysfunction-associated steatotic liver disease (MASLD) and liver fibrosis, and its predictive validity for both conditions remains unclear. Our results extend this understanding to MASLD and liver fibrosis, bridging a critical gap in the literature, we utilized data from the NHANES database (2017–2020) involving American participants, discovering a significant association between NHHR, MASLD, and liver fibrosis, indicating that NHHR can serve as a predictive marker for these conditions. This distinction is vital, as MASLD incorporates a broader spectrum of metabolic abnormalities, aligning with recent consensus definitions (11).

Metabolic syndrome, characterized by a cluster of interrelated risk factors, primarily involves abdominal obesity and insulin resistance (22, 23). Research has shown that in patients with metabolic syndrome, primarily exhibiting insulin resistance, increased circulating insulin levels may lead to enhanced synthesis and uptake of fatty acids by hepatic lipogenic cells, exacerbating triglyceride accumulation in the liver (24, 25). Ectopic fat accumulation in the liver further predisposes individuals to metabolic dysregulation, primarily presenting as dyslipidemia and insulin resistance (26). Additionally, a retrospective study indicated that NHHR offers superior diagnostic value for metabolic syndrome compared to commonly used metrics like apolipoprotein B/apolipoprotein A1 ratios (27). Thus, we focused on NHHR as a primary research target, revealing it to be a risk factor for MASLD. To further validate the relationship between NHHR and MASLD across different populations, we conducted subgroup analyses. Results in our research showed that NHHR as an independent risk factor for MASLD was significant in the majority of populations with a high prevalence of metabolic syndrome, encompassing older adults aged over 60, women, individuals without diabetes, those with hypertension, and those who are overweight or obese. This also confirms the general applicability of NHHR as a risk factor.

In the definition of NHHR, HDL-C represents HDL levels in the blood, while non-HDL-C primarily includes measurements of low-density, intermediate-density, and very low-density lipoprotein cholesterol (28). It is well established that synthesized triglycerides are transported out of the liver in the form of very low-density lipoproteins (VLDL) (29). Our study suggests that NHHR is a protective factor against liver fibrosis, which seemingly related to VLDL changes. We hypothesize that within a certain range, as NHHR increases, the relative content of VLDL rises, aiding in the reduction of abnormal intrahepatic fat accumulation. In addition, it is possible that individuals with higher NHHR may represent a population that has developed an adaptive response to hepatic steatosis. In some cases, the liver may be able to tolerate higher levels of fat accumulation without progressing to fibrosis due to enhanced metabolic flexibility or protective mechanisms that mitigate liver damage. Higher NHHR could be associated with a different inflammatory profile. For instance, individuals in the upper quartiles may have a lower degree of inflammation or a more favorable cytokine profile, which could protect against the progression to advanced fibrosis. This explains our findings that NHHR, as a measure of lipid changes, shows independent protective effects against liver fibrosis primarily in younger and normal-weight populations. Younger populations typically have a more robust immune response and lower levels of chronic inflammation compared to older individuals (30). Chronic inflammation is a significant driver of liver fibrosis, as it promotes the activation of hepatic stellate cells, which are responsible for collagen deposition in the liver. In normal-weight individuals, the absence of obesity-related inflammation may further contribute to a lower risk of fibrosis (31, 32). Additionally, our research found that in middle-aged, diabetic, hypertensive, and obese populations, NHHR serves as an independent risk factor for liver fibrosis. We believe this is related to the presence of insulin resistance in these groups, where circulating insulin inhibits the secretion of VLDL into systemic circulation through complex mechanisms such as the degradation of apolipoprotein B (6). Consequently, this leads to the abnormal accumulation of fat in the liver, which is engulfed by blood-derived macrophages (monocyte-derived macrophages, MoMFs) and forms foam cells (33, 34). The increase in MoMFs partially replaces hepatic macrophages (Kupffer cells, KCs) and indirectly activates KCs to release pro-inflammatory factors, subsequently stimulating the transformation of hepatic stellate cells (HSCs) into myofibroblasts, ultimately resulting in liver fibrosis (35, 36). Our findings suggest that NHHR-associated lipid imbalances may modulate this inflammatory cascade, offering a potential therapeutic target.

To further investigate the predictive value and optimal threshold of NHHR for MASLD and liver fibrosis, we constructed ROC curves and restricted cubic spline (RCS) regression models. The results indicate that the areas under the curve (AUC) for MASLD_*VCTE*_, advanced liver fibrosis VCTE, MASLD_*FLI*_, advanced liver fibrosis FIB-4 and advanced liver fibrosis BARD were 0.643 (95% CI 0.632–0.654), 0.554 (95% CI 0.538–0.569), 0.680 (95% CI 0.664–0.695), 0.677 (95% CI 0.637–0.716) and 0.510 (95% CI 0.499–0.521), respectively. Which means that the diagnostic value of NHHR for MASLD_*FLI*_ is superior to MASLD_*VCTE*_, advanced liver fibrosis VCTE, advanced liver fibrosis FIB-4, advanced liver fibrosis BARD. RCS models identified a U-shaped nonlinear relationship between NHHR and both MASLD. By recognizing the “U-shaped” relationship, healthcare providers can implement more effective monitoring and treatment strategies for patients with varying NHHR values. To our knowledge, our study is the first to demonstrate the correlation between NHHR, MASLD, and liver fibrosis, as well as to establish the feasibility for NHHR in predicting them through RCS regression and ROC analysis. Our research findings demonstrated strong robustness across different models and methodologies. The utilization of multiple statistical methods and models in this study not only enhanced the reliability of the conclusions but also provided a more in-depth level of analysis. By identifying NHHR as protective in metabolically healthy populations but harmful in high-risk groups, we highlight the importance of personalized risk stratification. Additionally, with lifestyle changes and an aging population, the prevalence of MASLD is rapidly increasing (37). Therefore, it is essential to identify the risk of MASLD at an early stage using reliable biomarkers. As a simple and cost-effective index, NHHR is comparable or even better than the complex index in the diagnostic performance of MASLD and fibrosis. Furthermore, our proposed link between NHHR, VLDL dynamics, and macrophage-driven fibrosis provides a framework for future studies. Given the ease of obtaining lipid parameters in clinical practice and the straightforward calculation of NHHR, which is significantly correlated with MASLD and liver fibrosis and demonstrates high predictive accuracy, we recommend that greater emphasis be placed on the evaluation of NHHR.

### Study strengths and limitations

This article has several advantages: (1) First, it is the first study to confirm the association between NHHR and both MASLD and advanced liver fibrosis in a large population. (2) The research includes both primary and supplementary analyses, making the conclusions more robust and reliable. (3) This study adjusted for potential covariates to reduce the influence of confounding factors, thereby clarifying the independent relationship between NHHR and MASLD as well as advanced liver fibrosis. (4) We not only employed conventional regression analysis to demonstrate the relationship between NHHR and the diseases but also utilized restricted cubic splines (RCS) and receiver operating characteristic (ROC) analysis to enrich our study. This multifaceted approach provides robust evidence of the relationship between NHHR and liver diseases from various angles.

Also, the current study has several limitations: (1) The diagnosis of MASLD has not yet been widely implemented in clinical practice, which may restrict the applicability of the results. (2) It must be acknowledged that some covariates in the data used in this study were not recorded or measured, thus it is not possible to completely exclude the influence of unmeasured or unknown confounding factors on the results. (3) This study only analyzed the predictive value of baseline NHHR for MASLD and liver fibrosis recorded during a single examination, and it does not confirm whether the continuous dynamic changes of NHHR during follow-up would outperform the baseline NHHR alone. However, our findings indicate that the NHHR recorded at a single time point provides good predictive value for the occurrence of MASLD and liver fibrosis, and its assessment is more economical and demonstrates better patient compliance compared to other invasive tests. (4) The current study population primarily consists of individuals from the United States, highlighting the need for future research involving diverse racial groups. (5) This study is a cross-sectional study, and it cannot establish a causal relationship between NHHR and MASLD as well as advanced Liver Fibrosis. (6) While our chosen methods provided useful insights, they may not capture all the complexities of the data. Some alternative methods, such as random forest, might align better with the experimental hypotheses. (7) Using secondary databases may inevitably encounter issues such as sampling bias, variable constraints, self-reported data and missing data in the data collection process.

## Conclusion

Our study concludes that NHHR is associated with the risk of MASLD and advanced liver fibrosis in US adults. Further prospective studies and randomized controlled trials are required to confirm our findings. Further investigation is also needed to explore the underlying mechanisms and potential therapeutic effects.

## Data Availability

The original contributions presented in this study are included in this article/Supplementary material, further inquiries can be directed to the corresponding author.
