# Supplementary material for: The relationship between the ratio of non-high-density lipoprotein cholesterol to high-density lipoprotein cholesterol (NHHR) and both MASLD and advanced liver fibrosis: evidence from NHANES 2017–2020
Source: Front Nutr. 2025 Feb 27;11:1508106. doi: 10.3389/fnut.2025.1508106 (PMC11903283; doi:10.3389/fnut.2025.1508106)
Supplement: Supplementary file 1 [file Table_1.docx]

| Variable | Imputation ratio |
| --- | --- |
| Age | 0% |
| Sex | 0% |
| Race | 0% |
| PIR | 13.4% |
| Education levels | 0% |
| Smoke status | 0% |
| Alcohol status | 0% |
| DM | 0% |
| Hypertension | 0% |
| ALT | 0.6% |
| AST | 0.9% |
| BMI | 0.9% |
| NHHR | 0% |
| Walk/bicycle time | 77% |
| Work activity time | 54.8% |
| Recreational activity time | 52% |
| WC | 3.8% |
| Physical activity total time | 25.9% |

Supplementary Table 1 The proportion of missing covariates.
