# Supplementary material for: The relationship between the ratio of non-high-density lipoprotein cholesterol to high-density lipoprotein cholesterol (NHHR) and both MASLD and advanced liver fibrosis: evidence from NHANES 2017–2020
Source: Front Nutr. 2025 Feb 27;11:1508106. doi: 10.3389/fnut.2025.1508106 (PMC11903283; doi:10.3389/fnut.2025.1508106)
Supplement: Supplementary file 2 [file Table_2.docx]

| Variable | Pre-imputation | imputation | P value |
| --- | --- | --- | --- |
| PIR | 2.67 | 2.69 | 0.452 |
| ALT | 21.63 | 21.64 | 0.952 |
| AST | 21.50 | 21.51 | 0.946 |
| Walk/bicycle time | 297.05 | 297.05 | 1 |
| BMI | 29.83 | 29.82 | 0.93 |
| WC | 100.70 | 100.78 | 0.744 |
| Work activity time | 1657.73 | 1657.73 | 1 |
| Recreational activity time | 415.31 | 415.31 | 1 |
| Physical activity total time | 1372.97 | 1372.97 | 1 |

Supplementary Table 2 Data difference analysis before and after imputation.
