# Supplementary material for: The relationship between the ratio of non-high-density lipoprotein cholesterol to high-density lipoprotein cholesterol (NHHR) and both MASLD and advanced liver fibrosis: evidence from NHANES 2017–2020
Source: Front Nutr. 2025 Feb 27;11:1508106. doi: 10.3389/fnut.2025.1508106 (PMC11903283; doi:10.3389/fnut.2025.1508106)
Supplement: Supplementary file 3 [file Table_3.docx]

|  | Model 1 | | Model 2 | | | Model 3 | | |  |
| --- | --- | --- | --- | --- | --- | --- | --- | --- | --- |
|  | OR(95%CI) | P value | | OR(95%CI) | P value | | OR(95%CI) | P value | |
| **NHHR** | 1.75(1.52, 2.01) | <0.0001* | | 1.89(1.34, 2.68) | 0.001* | | 1.80(1.23, 2.62) | 0.01* | |
| **Q1** | Ref | Ref | | Ref | Ref | | Ref | Ref | |
| **Q2** | 1.65(1.28, 2.13) | <0.001* | | 1.47(0.90, 2.40) | 0.11 | | 1.31(0.75, 2.30) | 0.31 | |
| **Q3** | 3.48(2.63, 4.59) | <0.0001* | | 4.65(2.96, 7.32) | <0.0001* | | 3.90(2.46, 6.17) | <0.0001* | |
| **Q4** | 6.49(4.68, 9.00) | <0.0001* | | 13.32(7.53, 23.54) | <0.0001* | | 11.66(6.56, 20.72) | <0.0001* | |
| **P for trend** |  | <0.0001* | |  | <0.0001* | |  | <0.0001* | |

**Supplementary Table 3 Association between NHHR and MASLD diagnosed by FLI.**

Model 1: Non-adjusted.

Model 2: Adjusted for age, sex, education level, race, PIR, PA, BMI, WC, smoke status and alcohol status.

Model 3: Adjusted for age, sex, education level, race, PIR, PA, BMI, WC, smoke status, alcohol status, ALT, AST, TG, LDL, HOMA-IR, DM and Hypertension.

*p＜0.05
