# Supplementary material for: The relationship between the ratio of non-high-density lipoprotein cholesterol to high-density lipoprotein cholesterol (NHHR) and both MASLD and advanced liver fibrosis: evidence from NHANES 2017–2020
Source: Front Nutr. 2025 Feb 27;11:1508106. doi: 10.3389/fnut.2025.1508106 (PMC11903283; doi:10.3389/fnut.2025.1508106)
Supplement: Supplementary file 4 [file Table_4.docx]

|  | Model 1 | | Model 2 | | | Model 3 | | |  |
| --- | --- | --- | --- | --- | --- | --- | --- | --- | --- |
|  | OR(95%CI) | P value | | OR(95%CI) | P value | | OR(95%CI) | P value | |
| **NHHR** | 0.78(0.54, 1.13) | 0.18 | | 1.04(0.70, 1.55) | 0.84 | | 0.85(0.60, 1.21) | 0.33 | |
| **Q1** | Ref | Ref | | Ref | Ref | | Ref | Ref | |
| **Q2** | 0.55(0.37, 0.83) | 0.01* | | 0.58(0.31, 1.06) | 0.07 | | 0.61(0.27, 1.38) | 0.21 | |
| **Q3** | 0.20(0.11, 0.36) | <0.0001* | | 0.20(0.08, 0.51) | 0.002* | | 0.32(0.13, 0.80) | 0.02* | |
| **Q4** | 0.42(0.18, 0.95) | 0.04* | | 0.82(0.30, 2.22) | 0.67 | | 0.44(0.17, 1.16) | 0.09 | |
| **P for trend** |  | 0.009* | |  | 0.363 | |  | 0.027* | |

**Supplementary Table 4 Association between NHHR and advanced liver fibrosis** **diagnosed by FIB-4.**

Model 1: Non-adjusted.

Model 2: Adjusted for age, sex, education level, race, PIR, PA, BMI, WC, smoke status and alcohol status.

Model 3: Adjusted for age, sex, education level, race, PIR, PA, BMI, WC, smoke status, alcohol status, ALT, AST, TG, LDL, HOMA-IR, DM and Hypertension.

*p＜0.05
