# Supplementary material for: The relationship between the ratio of non-high-density lipoprotein cholesterol to high-density lipoprotein cholesterol (NHHR) and both MASLD and advanced liver fibrosis: evidence from NHANES 2017–2020
Source: Front Nutr. 2025 Feb 27;11:1508106. doi: 10.3389/fnut.2025.1508106 (PMC11903283; doi:10.3389/fnut.2025.1508106)
Supplement: Supplementary file 5 [file Table_5.docx]

|  | Model 1 | | Model 2 | | | Model 3 | | |  |
| --- | --- | --- | --- | --- | --- | --- | --- | --- | --- |
|  | OR(95%CI) | P value | | OR(95%CI) | P value | | OR(95%CI) | P value | |
| **NHHR** | 0.99(0.96, 1.03) | 0.64 | | 0.81(0.75, 0.88) | <0.0001* | | 0.85(0.77, 0.94) | 0.004* | |
| **Q1** | Ref | Ref | | Ref | Ref | | Ref | Ref | |
| **Q2** | 1.24(1.04, 1.47) | 0.02* | | 1.01(0.77, 1.32) | 0.96 | | 1.14(0.85, 1.52) | 0.34 | |
| **Q3** | 1.31(1.11, 1.54) | 0.002* | | 0.81(0.68, 0.95) | 0.01* | | 1.22(0.95, 1.57) | 0.10 | |
| **Q4** | 1.09(0.95, 1.26) | 0.22 | | 0.57(0.43, 0.74) | <0.001* | | 0.79(0.56, 1.12) | 0.17 | |
| **P for trend** |  | 0.224 | |  | <0.0001* | |  | 0.141 | |

**Supplementary Table 5 Association between NHHR and advanced liver fibrosis** **diagnosed by BARD.**

Model 1: Non-adjusted.

Model 2: Adjusted for age, sex, education level, race, PIR, PA, BMI, WC, smoke status and alcohol status.

Model 3: Adjusted for age, sex, education level, race, PIR, PA, BMI, WC, smoke status, alcohol status, ALT, AST, TG, LDL, HOMA-IR, DM and Hypertension.

*p＜0.05
